# Dielectric Spectroscopy and Application of Mixing Models Describing Dielectric Dispersion in Clay Minerals and Clayey Soils

**DOI:** 10.3390/s20226678

**Published:** 2020-11-22

**Authors:** Juan D. González-Teruel, Scott B. Jones, Fulgencio Soto-Valles, Roque Torres-Sánchez, Inmaculada Lebron, Shmulik P. Friedman, David A. Robinson

**Affiliations:** 1 Department of Automatics, Electrical Engineering and Electronic Technology, Technical University of Cartagena, 30202 Murcia, Spain; pencho.soto@upct.es (F.S.-V.); roque.torres@upct.es (R.T.-S.); 2 Department Plants, Soils and Climate, Utah State University, Logan, UT 84322, USA; scott.jones@usu.edu (S.B.J.); davi2@ceh.ac.uk (D.A.R.); 3 UK Centre for Ecology and Hydrology, ECW, Bangor LL572UW, UK; inmbin@ceh.ac.uk; 4 Institute of Soil, Water and Environmental Sciences, Agricultural Research Organization, The Volcani Center, Rishon LeZion 7505101, Israel; vwsfried@volcani.agri.gov.il

**Keywords:** dielectric sensors, dielectric spectroscopy, clayey soil, mixing model, Maxwell–Wagner relaxation, two-phase modeling, soil moisture

## Abstract

The number of sensors, ground-based and remote, exploiting the relationship between soil dielectric response and soil water content continues to grow. Empirical expressions for this relationship generally work well in coarse-textured soils but can break down for high-surface area and intricate materials such as clayey soils. Dielectric mixing models are helpful for exploring mechanisms and developing new understanding of the dielectric response in porous media that do not conform to a simple empirical approach, such as clayey soils. Here, we explore the dielectric response of clay minerals and clayey soils using the mixing model approach in the frequency domain. Our modeling focuses on the use of mixing models to explore geometrical effects. New spectroscopic data are presented for clay minerals (talc, kaolinite, illite and montmorillonite) and soils dominated by these clay minerals in the 1 MHz–6 GHz bandwidth. We also present a new typology for the way water is held in soils that we hope will act as a framework for furthering discussion on sensor design. We found that the frequency-domain response can be mostly accounted for by adjusting model structural parameters, which needs to be conducted to describe the Maxwell–Wagner (MW) relaxation effects. The work supports the importance of accounting for soil structural properties to understand and predict soil dielectric response and ultimately to find models that can describe the dielectric–water content relationship in fine-textured soils measured with sensors.

## 1. Introduction

An empirical polynomial expression was proposed by Topp et al. [1] to account for the sample scale of soil apparent dielectric response (*K*) to soil volumetric water content (*θ_V_*), measured using time domain reflectometry (TDR):

(1) $$\theta_{V}=\left(-530+292\times K-5.5\times K^{2}+0.043\times K^{3}\right)\times 10^{-4}$$

The vast majority of soil moisture dielectric sensors use this equation to provide a water content value from the measured apparent permittivity. The equation proved highly successful in a wide range of coarse and medium-textured soils. However, some materials clearly deviate from this relationship, pointing to vermiculite data, and subsequent work has demonstrated that clayey mineral soils also deviate from this relationship, thus limiting the accuracy of soil dielectric sensors. In a personal communication, Clarke Topp postulated that the reason the Canadian clayey soils adhered to Equation (1) might have been because the particles were clay-sized but not dominated by expansive clay minerals. The dielectric response of clayey mineral soils measured using TDR has often been shown to fall below Equation (1) [2,3,4]. It is worth noting that the opposite has been observed for measurements lower in the radio frequency range, e.g., 20 MHz [5]. The work of Hallikainen et al. [6] and Dobson et al. [4] was important in proposing that “bound” water, which is rotationally hindered by adsorption to surfaces, could explain the lower dielectric responses of high-clay content (larger specific surface) soils at high frequencies. The theory worked well for the results they presented. However, alternative work in geophysics and soil science demonstrated that deviation from Equation (1) could also occur due to changes in bulk density [3], the geometry of the particles contained in a porous medium [7,8] or the geometrical configuration of the water phase [7,8,9,10,11], due to aggregation, for example [12], where the pore network connectivity, dependent on the phase configuration, plays an important role in the bulk dielectric response. This opens the question of which phenomena, “bound” water or geometrical structure, affect the dielectric response, to what extent and under what conditions. As Wagner and Scheuermann [13] point out, “The results of the study by Blonquist et al. [12] indicate that further work is necessary to quantify and define the relative contributions of both ‘bound’ water and confined water in dependence of material microstructure to the bulk electromagnetic response”. Here, we contribute to this exploration, we briefly discuss evidence for “bound” water and geometrical effects and propose a typology for discussing these effects before considering the dielectric response in the frequency domain.

Soils are well known to contain “bound” water, often estimated as the amount of hygroscopic water [14], though to what extent this impacts dielectric measurements remains poorly understood and open to investigation [15,16,17,18,19,20]. The impact of “bound” water on dielectric measurements has support based on experimental evidence mostly from work conducted on mineral and non-mineral surfaces, e.g., in food [21]. Thorp [22] proposed that the first monolayer of water vapors adsorbed on silica gel had a dielectric value of ~41 and that the second monolayer was ~66. Bockris et al. [23], studying a metal surface, proposed a value of 6 for the first immobile water layer, close to the high-frequency dielectric value, and 32 for the second layer before rising to that of free water in the third layer (~1 nm). The dielectric behavior of water at smectite clay mineral surfaces was reviewed by Sposito and Prost [24]. They reported that water adsorbed on Na- and K-saturated smectite surfaces had a similar relaxation time to bulk water, whereas it was increased in clays saturated by divalent cations, such as Ca and Mg. Nevertheless, in both cases, larger values of the Cole–Cole [25] parameter, describing the spread in relaxation frequency, compared with that of bulk water, revealed a broad variety of molecular environments in adsorbed water at very low water contents. This is graphically shown in Supplementary Material Figures S1 and S2.

One of the challenges with determining the behavior of water at surfaces, particularly the dielectric behavior, is the extreme difficulty in measurement. New measurement methods continue to offer insight into the behavior of water near interfaces [26,27,28]. Teschke et al. [21] used atomic force microscopy to measure water dielectric profiles on mica. As with others, they found a cation charge dependence, however they also found that bulk dielectric values were not obtained until ~10 nm from the surface, much more than with other materials.

Molecular dynamics simulations have also provided evidence of alteration of bulk water properties near surfaces. Mulla et al. [29] modeled an uncharged silica surface and proposed that “surfaces induce considerable hindrance in the rotational motion of water molecules located as far as 10 Å (1 nm) from the surfaces.” They also pointed out that the dipole orientations confined between layers are hindered, despite the surfaces being uncharged and without cations. The structural configuration of the confined water alters the dipole orientation rather than that associated with electrostatic forces due to ion hydration or negatively charged surfaces. Bonthuis et al. [30] opined that “the question of whether the decrease of the dielectric profile reflects ionic or rather intrinsic water properties is still subject to debate”. Mechanisms for the reduction of the dielectric value at the molecular scale include electrostatic mechanisms, such as the dielectric decrement associated with a high concentration of ions and their hydration [31], or dielectric saturation due to proximity to charged interfaces and a high surface electric field strength [32]. Molecular ordering, hydrogen bonding disruption and orientation of water are also cited as mechanisms that can cause water to deviate from bulk properties. Water has been shown to deviate from bulk properties when confined or caged in spheres or nanotubes with radii of <5 Å, for example [33,34]. In addition, the nature of the surface [30], whether it is hydrophilic, like most soil minerals, or hydrophobic, like many soil organic materials, is also important and affects the water structure. Research into the behavior of confined water is reviewed by Thompson [35], who also acknowledges that we still have much to learn regarding the dielectric behavior of water near surfaces. It must also be borne in mind that much of this research is on the static dielectric response of water.

Moving away from molecular phenomena, we now consider evidence for the effect of macro-scale geometry on the dielectric response; this includes particle shape, orientation, size distribution and phase configuration. Jones and Friedman [7] demonstrated using mica, without “bound” water, that the aspect ratio of the particles and their alignment with the electrical field could result in substantial amplification or damping of the dielectric response, depending on the particle orientation with the applied field. Particle size distribution has also been shown to reduce the permittivity response as the smaller particles perturb the field around the larger particles [36]. Robinson and Friedman [11] demonstrated that packings of monosize spheres behave approximately according to the Maxwell Garnett (MG) mixing formula, which assumes that a particle only sees a fluid dielectric background, while packings with increasing width of particle size distribution diverge, converging on the Sen, Scala and Cohen [37] self-similar model. This differential effective medium approximation (DEMA) assumes a sequential mixing of particles and fluid to form an effective background that the particle experiences and is most suited to describing the dielectric response of low-porosity sediments (0.2–0.25 cm^3^ cm^−3^).

The configuration of the solid, water and air phases is also significant. This was demonstrated by Friedman [10] for spherical, and by Jones and Friedman [7] for ellipsoidal particles. Phase configuration is important because of the asymmetric dielectric response of two or more phases, as demonstrated by the Maxwell Garnett [38] model. For a 50% mixture of spheres embedded in a host background, the effective dielectric value is 35.6 if the inclusion is solid (ε ~ 5) and surrounded by a background of water (ε ~ 80), which drops to 15.7 if the configuration is changed and the solid becomes the background. This is also demonstrated in aggregated media both through measurement [12] and modeling [39], producing lower effective dielectric responses than might be expected based on the water saturation percentage.

In order to contribute to this debate, we propose abandoning the term “bound water” and adopting a typology to more accurately describe the state of water in soils, relevant to dielectric measurements, to help frame the discussion around investigating these phenomena:

- Free water, which exhibits bulk properties of a continuous background phase.
- Macroscopically confined water that exhibits bulk properties but is confined by the configuration of the solid, water and air phases, e.g., in aggregates or foams, as a discontinuous inclusion phase.
- Microscopically confined water where the dielectric response of the water is modified, for example, by:
  ○ Extrinsic electric fields, including dipole forces, charged surfaces or hydration around ions.
  ○ Extrinsic geometry, causing structural alteration due to being trapped, caged or structurally modified by proximity to a surface.

The ultimate scientific aim is to distinguish the extent to which each of these mechanisms can influence the effective dielectric response of a porous medium.

**Figure 1:**
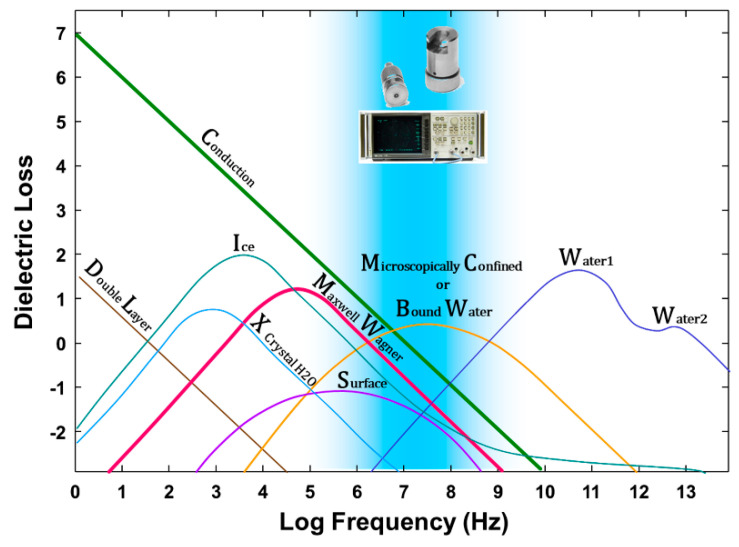
Contributors to dielectric loss in wet porous media covering a large frequency spectrum. Mechanisms include conduction (ionic conductivity), charged double layer, crystal water relaxation, ice relaxation, Maxwell–Wagner relaxation, surface conductivity, microscopically confined water relaxation; principle free water relaxation (Water 1), second free water relaxation (Water 2) (after Hasted [40], p. 238).

The discussion thus far has focused on the “static” dielectric response, but most sensors operate in the radio frequency–microwave region of the frequency domain, which is where we need new understanding. Dielectric dispersion is the dependence of the dielectric response of a material on the frequency of an applied electric field. Dielectric materials are subject to a range of processes in the frequency domain that result in dielectric loss that may also impact the real part of the dielectric response. Figure 1 illustrates a summary of possible contributions to dielectric loss within moist heterogeneous systems, as presented by Hasted [40]. These relaxation mechanisms affect the imaginary part of the dielectric response, especially at lower frequencies, and some of them may also affect the real part [18]. This highlights the complexity in explaining the causes of the different dielectric behaviors between the real and imaginary parts and among different materials. In the radio frequency–microwave region of interest, electrical conductivity (C), Maxwell–Wagner polarization (MW) and microscopically confined water (B) are all relevant processes. The figure shows the overlap between Maxwell–Wagner (MW) losses, mostly due to the interaction of ionic conduction (C) in confined geometries, and the zone where microscopically confined water (B) relaxation is likely to occur based on measurements in foodstuffs.

In the case of fluids, we have a reasonable understanding of the frequency domain behavior in the microwave region, often empirically described by the Debye, Cole–Cole, Cole–Davidson and Havriliak–Negami formulas [25,41,42,43,44]. Dielectric mixing models can be transformed into frequency-domain dielectric models by inserting the frequency dependence of water into the model, properly described by the models cited above. However, dielectric spectra of rocks with ionic conductivity [37,45] or soils [46] show that the dispersion behavior is a complex interplay between different phenomena linked to the geometry [45,46,47], the water structure, due to macro-scale or micro-scale confinement [21,48,49], and the contrast in permittivity and conductivity in the interphase between the mixture phases [50,51,52,53,54]. The growth in dielectric spectroscopy and reduction in the cost of equipment has opened up the opportunity to obtain better frequency-domain dielectric measurements [55]. However, challenges remain to unravel the interplay between dielectric loss processes described above in order to fully realize the potential of electrical measurements.

This paper does not attempt to address all these questions, however, we seek to make some progress on developing both the data and models which take us in this direction and identifying unexpected dielectric responses that need to be explained. The objectives of this work are therefore to:

1. Measure the dielectric dispersion of well-defined clay minerals and associated clayey soils, and to compare these with other unsaturated porous media where either macro- or microscopically confined water should be dominant.
2. Use simple models to test the predictive capability of the geometrical modeling approach for water-saturated dispersive clayey soils.
3. Compare the dispersive data for unsaturated clayey soils with the mixing model bounds we might expect for coarse granular materials.

## 2. Materials and Methods

### 2.1. Clay Minerals

The minerals and reference clay minerals used in this study are those presented by Robinson [56] and described in detail there. Briefly, the minerals were obtained from a number of different sources: talc (1:1 clay mineral structure) came from Aldrich Chemical Co. Inc., Milwaukee, WI, and the other clay minerals, kaolin (1:1 structure), Silver Hill illite (2:1 structure) and sodium bentonite (2:1 structure), came from The Soil Clay Minerals Repository, University of Missouri, Columbia (properties, including hygroscopic water, can be found in Supplementary Material Tables S1 and S2). Talc was chosen as a mineral with a layered silicate structure but no surface charge and low surface area in contrast to the other clay minerals, with increasing layer charge and hygroscopic water content. Clay minerals solid particles permittivity was obtained using the immersion method described by Robinson and Friedman [57]. The values obtained were: talc, 5.3; kaolinite, 5.1; illite, 5.8; and smectite, 5.5 [56].

### 2.2. Clayey Soils

Soils were obtained from the United States Department of Agriculture (USDA). The Cecil soil series is a Fine, kaolinitic, thermic Typic Kanhapludult, which occurs mostly in the Piedmont region of the Southeastern United States. The Blount soil is a Fine, illitic, mesic Aeric Epiaqualf occurring in the till soils of the Midwestern United States. The Okoboji soil is a Fine, smectitic, mesic Cumulic Vertic Endoaquoll. They are very poorly drained soils formed in alluvium or lacustrine sediments from Iowa. The water used to wet the samples had an electrical conductivity (EC) of 0.03 S m^−1^. The solution was extracted using a 2:1 dilution in deionized water at 25 °C and the EC of the extracts measured was: Cecil, 0.0366; Blount, 0.0385; and Okoboji, 0.0970 S m^−1^. Since the saturated gravimetric water content of clayey soils is generally assumed to be ~0.5 g g^−1^, the 2:1 dilution EC above was multiplied by 4 to obtain the water-saturated *EC_w_* used in fitting the dielectric mixing models. The bulk DC conductivity (σ_aDC_) of water-saturated clayey soils samples was also determined, as reported in the Supplementary Material (Figure S3).

### 2.3. Vector Network Analyzer (VNA) Measurements

The dielectric properties of the clay minerals and clayey soils were measured as a function of frequency with a VNA (Model 8753B, Hewlett-Packard, Palo Alto, CA, USA) using an open-ended coaxial dielectric probe (HP 85070B High-Temperature Dielectric Probe Kit, Hewlett-Packard, Palo Alto, CA, USA) fitted with a 3.17 cm^3^ sample holder. Measurements spanned a 1 MHz–6 GHz bandwidth. The VNA was calibrated using the HP 85033C 3.5 mm Calibration Kit (Hewlett-Packard, Palo Alto, CA, USA) which includes the open, short and load (50 Ω) standards. Network analyzers can measure the reflection (one port) and transmission (two ports) characteristics of the medium of interest using a broad bandwidth signal [58]. For this study, reflection measurements were performed to obtain the dielectric properties of the clays and clayey soils. In order to obtain the complex permittivity from the reflection coefficient (S11) values provided by the VNA, the dielectric probe was calibrated using air, deionized water and a shorting block as standards, together with the calibration software supplied with the probe, following the steps reported in the probe manual [59].

Air-dried clay and clayey soil samples (<250 µm) were wetted with a fine spray, mixed thoroughly to ensure a uniform distribution of water throughout the sample and left to equilibrate for 48 h. The samples were carefully packed in the sample holder to achieve a loose packing. A measurement was made and then the soil was vibrated and compressed. The next dielectric measurement was made. The amount of moist soil added was recorded at all times. This process was repeated until a tight packing was achieved. Hence, a range of bulk densities and moisture contents were achieved. The sample holder was packed with up to four different bulk densities for each soil volumetric water content, the latter determined by oven drying. Measurements were conducted in a temperature-controlled room at ~25 °C.

## 3. Theory and Modeling

Perhaps the most widely used model for describing the effective permittivity (*ε_eff_*) of a two-phase mixture of dielectric spheres in a uniform dielectric background is the MG [38] mixing equation [60], given as

(2) $$\varepsilon_{eff}=\varepsilon_{e}+3f\varepsilon_{e}\left(\frac{\varepsilon_{i}-\varepsilon_{e}}{\varepsilon_{i}+2\varepsilon_{e}-f(\varepsilon_{i}-\varepsilon_{e})}\right)$$

where *f* is the volumetric fraction of the solid inclusions (=1 − *ϕ*, where *ϕ* is the porosity), *ε*_e_ is the permittivity of the background (water or air) and *ε*_i_ is the permittivity of the granular inclusions. The model assumes that the inclusions and their respective electrical fields are non-interacting, which is an invalid assumption for densely packed granular materials. This interaction effect has been studied theoretically for periodic cubic lattices of spheres [61] and demonstrated experimentally for these cubic lattices [36].

Bruggeman [62] previously and Hanai [50] and Sen et al. [37] later set out to develop a DEMA which would describe the permittivity of saturated porous rocks, approaching the behavior of densely packed granular materials. Instead of assuming that a particle “sees” a fluid background, the background is developed using sequential mixing of particles into the water phase so that a spherical particle “sees” a background with effective dielectric properties:

(3) $$\left(\frac{\varepsilon_{i}-\varepsilon_{eff}}{\varepsilon_{i}-\varepsilon_{e}}\right){\left(\frac{\varepsilon_{e}}{\varepsilon_{eff}}\right)}^{1/3}=\phi$$

This is known in the literature as the asymmetric Bruggeman or Sen formula ([50] derived for complex permittivities). This model therefore takes into account the field interaction between particles, since it is based on an infinitesimal sequential mixing of solid into the fluid background, which is then used as the background for the next mixing step [37]. Equations (2) and (3) are therefore expected to form upper and lower bounds for mixtures of spherical granular materials. Experimentally, this was demonstrated by Robinson and Friedman [11], where the effective permittivity migrated from close to Equation (2) for monosize materials to Equation (3) as the particle size distribution widened.

Sen’s model, when extended for complex electromagnetic properties and ellipsoidal particles, is often found in the literature as the Hanai–Bruggeman, Bruggeman–Hanai–Bussian, Maxwell–Wagner–Bruggeman–Hanai (MWBH) or Bruggeman–Hanai–Sen equation [63], and for randomly oriented ellipsoids of a revolution (spheroids) [64,65], it reads

(4) $$\phi \;=\;{\left(\frac{\varepsilon_{e}^{*}}{\varepsilon_{eff}^{*}}\right)}^{3d}\left(\frac{\varepsilon_{i}^{*}-\varepsilon_{eff}^{*}}{\varepsilon_{i}^{*}-\varepsilon_{e}^{*}}\right){\left[\frac{\varepsilon_{e}^{*}\left(1+3N^{b,c}\right)+\varepsilon_{i}^{*}\left(2-3N^{b,c}\right)}{\varepsilon_{eff}^{*}\left(1+3N^{b,c}\right)+\varepsilon_{i}^{*}\left(2-3N^{b,c}\right)}\right]}^{K}$$

where the asterisk * denotes complex values and

$$d=N^{b,c}\frac{\left(1-2N^{b,c}\right)}{\left(2-3N^{b,c}\right)}$$

$$K=\frac{2{\left(3N^{b,c}-1\;\right)}^{2}}{\left(2-3N^{b,c}\right)\left(1+3N^{b,c}\right)}$$

The effect of particle geometry is modeled using depolarization factors (*N^i^*) that describe the extent to which the inclusion polarization is reduced according to its shape and orientation with respect to the applied electrical field. The depolarization factors for a spheroid (ellipsoid with radii *a*, *b*, *c*, *a ≠ b = c*) with an aspect ratio of (*a/b*) can be approximated by the empirical function given by Jones and Friedman [7]:

(5) $$N^{a}=\frac{1}{1+1.6\left(a/b\right)+0.4{\left(a/b\right)}^{2}}\;N^{b}=N^{c}=0.5\left(1-N^{a}\right)$$

The depolarization factors for a sphere where *a*, *b* and *c* are all equal in length are *N^a^*^,*b*,*c*^ = 1/3, 1/3, 1/3, for thin disks *N^a^*^,*b*,*c*^ = 1, 0, 0 and for long needles *N^a^*^,*b*,*c*^ = 0, 0.5, 0.5. Friedman and Robinson [66] found that a value of (*a/b*) equal to 0.466 was representative of quartz sand grains.

Sihvola and Kong [67] proposed a unified mixing rule to account for intergrades by introducing an apparent dielectric value, *ε*_α_, in the model, which was defined as “the permittivity which an inclusion ‘feels’ in its surroundings in the mixture” [60]. It is expected to lie somewhere between the value of the bathing fluid, *ε_e_* (i.e., water or air) and *ε_eff_*. The model expression extended for complex values can be expressed as

(6) $$\varepsilon_{eff}^{*}=\varepsilon_{e}^{*}+\left\{\frac{\left[\sum_{i=a,b,c}\frac{f(\varepsilon_{i}^{*}-\varepsilon_{e}^{*})\varepsilon_{\alpha }^{*}}{3\left[\varepsilon_{\alpha }^{*}+N^{i}(\varepsilon_{i}^{*}-\varepsilon_{e}^{*})\right]}\right]}{\left[1-\sum_{i=a,b,c}\frac{fN^{i}(\varepsilon_{i}^{*}-\varepsilon_{e}^{*})}{3\left[\varepsilon_{\alpha }^{*}+N^{i}(\varepsilon_{i}^{*}-\varepsilon_{e}^{*})\right]}\right]}\right\}\;\varepsilon_{\alpha }^{*}=\varepsilon_{e}^{*}+\alpha (\varepsilon_{eff}^{*}-\varepsilon_{e}^{*})$$

The heuristic parameter, *α* (also termed “the self-consistency” parameter [60]), can be considered physically as representing a polarization resulting from the close packing of the particles. A value of *α* = 0 results in the MG formula (Equation (2)) and other well-known models result as *α* increases. The equivalence with the well-known symmetric effective medium approximation (Polder and van Santeen [68]) (PVS) depends upon the depolarization factors (*α^i^* = 1 − *N^i^*), giving rise to a different heuristic parameter for each principal axis and resulting in a single value of *α* = 2/3 for spheres. A value of *α* = 1 stands for the coherent potential approximation, assuming the inclusions “feel” around them a background of *ε_eff_*. Friedman and Robinson [66] found that a value of *α* = 0.2 accounted for the neighboring particle effects based on measurements in coarse, densely packed granular materials. Experimental evidence has further shown that this value does change depending on the packing of monosize spheres. Values of *α* ranged between 0.2 for the random packing of monosize spheres and 0.323 for the simple cubic periodic lattice [36].

The mixing models described provide the static dielectric response, however, they can be extended to the frequency domain by incorporating the frequency-dependent dielectric values of the constituents. The dielectric response of soil solid particles remains constant in the microwave region, so a model for the water is required. For some polar materials like water in the microwave frequency range, the dielectric dispersion stemming from orientational polarization can be mathematically described by the classical Debye equation [43]:

(7) $$\varepsilon^{*}\;=\varepsilon_{\inf}+\frac{\varepsilon_{s}-\varepsilon_{\inf}}{1+j\omega \tau }$$

where *ω* (rad s^−1^) is the electromagnetic angular frequency, *ε_s_* is the low-frequency limit of the dielectric value, *ε_inf_* is the high-frequency limit of the dielectric value, practically at a frequency high enough that the molecules cannot respond to the applied field (re-orient) and create polarization, and *τ* is the relaxation time (s), characterizing the time it takes the molecule dipoles to revert to their original random orientation after the external field is removed.

An in-depth discussion of the structure of water and the different models is beyond the scope of this paper and the reader is referred to Hasted [40] or the concise and updated review by Kaatze [69]. For distilled water at 25 °C, the Debye model parameters are *ε_s_* = 78.36, *ε_inf_* = 5.2 and *τ* = 8.27·10^−12^ s [70]. As the frequency increases, the real part, *ε*′, varies from its static value, 78.36, towards that of high frequencies, in a sigmoidal pattern, passing through an inflection point at the relaxation frequency of 19.25 GHz (*f_rel_* = 1/2πτ). The imaginary part, *ε*″, is zero for the static and infinite frequency states, and has a bell shape (symmetric when *ω* is plotted on a log scale), with its maximal value, 36.58 ((*ε_s_* − *ε_inf_*)/2), at the relaxation frequency.

To obtain the dielectric response of porous media in a saturated state as a function of measurement frequency, *ε_e_* in Equations (2)–(4) or (6) can be replaced by the Debye model for water. These models form the basis for testing geometrical effects on the frequency-domain dielectric response, onto which there is the potential to explore other phenomena. Based on soil solution measurements, the water phase of the studied water-saturated soils was considered as a conductive medium. To account for the conductivity effects on the effective dielectric response of soils, we added a term of conductivity to the imaginary part of the Debye model for the water phase, so that Equation (7) was extended to

(8) $$\varepsilon_{}^{*}=\varepsilon_{\inf}+\frac{\varepsilon_{s}-\varepsilon_{\inf}}{1+j\omega \tau }-j\frac{\sigma_{wDC}}{\omega \varepsilon_{0}}$$

where *σ_wDC_* is the *EC_w_* (*ω* ~ 0) of the solution and *ε_0_* is the permittivity of the vacuum (8.854 × 10^−12^ F m^−1^).

## 4. Results and Discussion

### 4.1. Clay Minerals and Clayey Soils Dispersion

**Figure 2:**
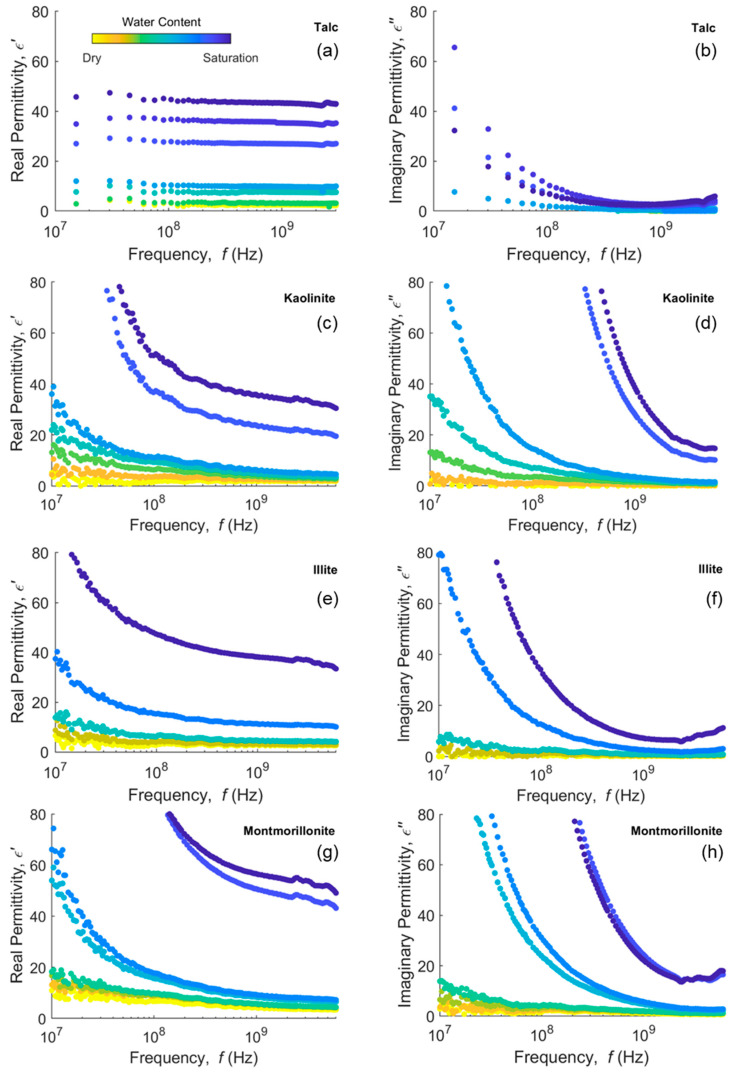
Real (left column) and imaginary (right column) permittivity measurements in (**a**,**b**) talc, (**c**,**d**) kaolinite, (**e**,**f**) illite and (**g**,**h**) montmorillonite clays at different water contents in a 10 MHz–6 GHz (10 MHz–3 GHz for talc) frequency bandwidth.

**Figure 3:**
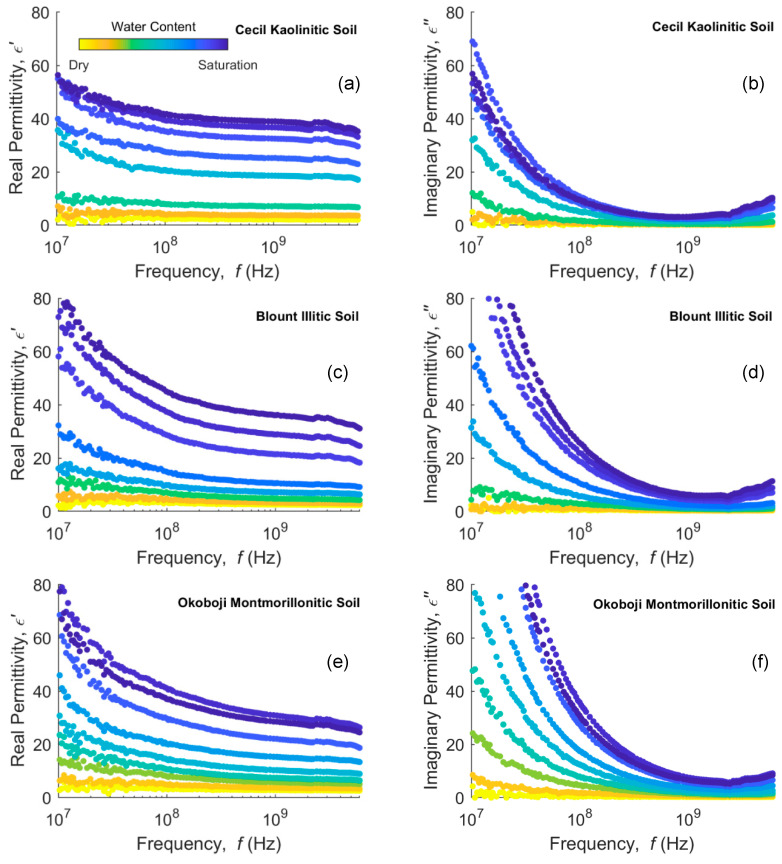
Real (left column) and imaginary (right column) permittivity measurements in (**a**,**b**) Cecil kaolinitic, (**c**,**d**) Blount illitic and (**e**,**f**) Okoboji montmorillonitic clayey soils at different water contents in a 10 MHz–6 GHz frequency bandwidth.

Data from VNA measurements of complex permittivity in clays and clayey soils for a range of water content are presented in Figure 2 and Figure 3, respectively. Clays and clayey soils subplots in these figures are ordered from 1:1 clays at the top to 2:1 clays at the bottom. With this ordering, we expect the least dispersion at the top, increasing down the subplots. As expected, both real and imaginary parts of the permittivity are higher as the water content increases.

In Figure 2a, the real permittivity of talc shows almost no dispersion in the response curves. The imaginary permittivity dispersion in Figure 2b corresponds to increases in the bulk EC as the water content increases. Kaolinite, a similar 1:1 mineral, but with surface charge, shows distinct dispersion in the real permittivity (Figure 2c), increasing at lower frequencies (<10^8^ Hz). Illite shows a similar response in Figure 2e,f, while montmorillonite exhibits the strongest dispersion (Figure 2g,h) for the real permittivity. While kaolinite and illite slightly level out at >10^8^ Hz, montmorillonite still has distinct curvature in the real permittivity throughout the frequency range. Real and imaginary permittivities of water-saturated and nearly water-saturated (kaolinite, ~0.32–0.41 m^3^ m^−3^; illite, ~0.55 m^3^ m^−3^; montmorillonite, ~0.84–0.88 m^3^ m^−3^) dispersive clays are substantially higher between 10^8^ and 10^9^ Hz, even exceeding the permittivity of pure water in some cases. Since this frequency range is common for many water content sensors, it is important to know the measurement frequency and sensor response in dispersive clayey soils in order to accurately relate dielectric response to water content.

Soils presented in Figure 3 show similar but less pronounced behavior, as expected, given that they are mixtures of clay minerals with other minerals and organic matter particles. The kaolinite Cecil soil (a,b) shows the least dispersion of the three soils, with the Blount (c,d) and Okoboji (e,f) being similar with more dispersion. The real permittivity does not necessarily increase in order of increasing water content and some responses of, e.g., water-saturated Okoboji soil (*θ_V_* = 0.56), can have a higher dielectric response at a lower water content (*θ_V_* = 0.53). This is consistent with the context in which interfacial polarization occurs, since in unsaturated soils, there are solid–water and air–water interfaces that contribute to the MW effect and thus the intensity of dispersion is not expected, in principle, to increase monotonically with water content.

Talc and kaolinite have the same basic 1:1 structure and little or no microscopically confined water, as measured by that adsorbed at 20% RH (Table S2), the surface charge being the main difference. Thus, we see that kaolinite responds in a very different way to talc, exhibiting dispersion for the real part of the dielectric permittivity. Unlike talc, kaolinite particles have charge and adsorbed cations. This likely leads to multiple polarization phenomena, with the main contributions of the surface conductivity and the interfacial polarization (MW effect), as a consequence of the mismatch between the solution and solid particle conductivities and permittivities, that may account for the enhanced dielectric response.

### 4.2. Modeling of Water-Saturated Clay Mineral Soils

**Figure 4:**
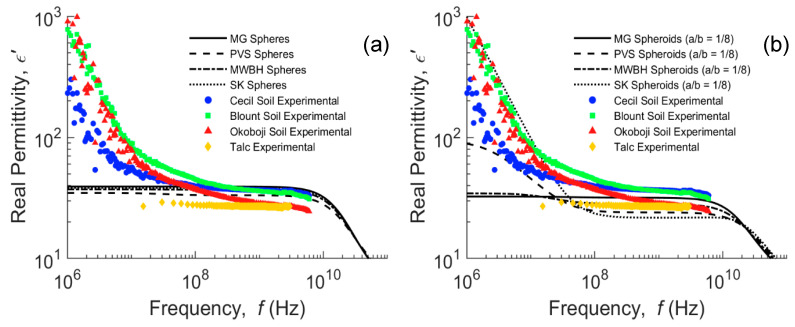
Real permittivity of Cecil, Blount and Okoboji water-saturated samples and unsaturated talc, and frequency-domain representation of Maxwell Garnett (MG), Polder van Santen (PVS), Maxwell–Wagner–Bruggeman–Hanai (MWBH) and Sihvola–Kong (SK) models for spherical (**a**) and oblate spheroidal (**b**) inclusions.

In Figure 4, we attempt to simulate the real dielectric of water-saturated soils (talc is unsaturated but at a similar water content of about 0.56 m^3^ m^−3^). We applied the Sihvola–Kong (SK) two-phase model (Equation (6)) with dielectric dispersion for the water phase (Equation (8)), where *σ_wDC_* = 0.2 S m^−1^, broadly in the middle of the three soils (Cecil = 0.14; Blount = 0.16; Okoboji = 0.40 S m^−1^), *α* = 0.2 and the particle shape was assumed to be spheres (Figure 4a) or oblate spheroids (Figure 4b). The electrically conductive water is considered as the background and the soil particles as non-conductive solid inclusions with a permittivity of 5. Similarly, MG (Equation (6) with α = 0) and PVS (Equation (6) with *α^i^* = 1 − *N^i^*) for spheroids and complex permittivities, and MWBH (Equation (4)) models are also presented in the frequency domain. The three dispersive soils show similar responses below 10^9^ Hz. The Okoboji soil dielectric response is distinctly lower in the higher frequencies (>10^8^ Hz) compared to the other two soils. The permittivity of talc exhibits a relatively dispersion-free response in comparison, a response that would also be expected in a sand, for example.

In Figure 4a, the simulated permittivity using the four different models is presented for spherical particles, none of which adequately account for the dielectric dispersion exhibited by the measurements below 10^8^ Hz. The particle aspect ratio, translated through *N^i^* in Equation (6), was adjusted to represent inclusion aspect ratios from spheres to oblate spheroids, although only that of *a/b* = 1/8 is presented in Figure 4b. The MG model assumes that the inclusions and their respective electrical fields are non-interacting, which is a valid assumption for diluted mixtures [36], constituting an upper bound for water-saturated soils. Nonetheless, this non-interaction is likely to explain the inability of the model to describe the MW dispersion. The DEMA in its different extensions, such as the MWBH formula, has proved very successful in the high-frequency limit (ω → ∞) [63], especially for media of broad particle size distribution [11,71,72]. However, its suitability in describing the interfacial polarization in water-saturated porous media below 10^8^ Hz is limited. The DEMA is based on an infinitesimal sequential mixing of solid into the fluid background, which corresponds to an “infinitely wide” particle size distribution, i.e., a fractal granular medium. This means that the background phase connectivity is never interrupted, regardless of the porosity and the particle shape and orientation. In contrast, in fine-grained soils dominated by clay minerals, as the shape of the solid particles tends to be a thin disk, their ability to form barriers that interrupt the continuity of the conductive aqueous phase, where also charge accumulates, increases. This gives rise to portions of confined electrically conductive water, which are separated by assemblies of solid particles with a different conductivity and permittivity, not contributing to the in-phase conductivity of the medium, but increasing the dielectric polarization [37]. This is likely the cause for the MWBH model not being able to explain the dispersion shown by all soils. Evidence of the emergence of a percolation threshold as we approach disk-shaped particles supports this hypothesis [73], and can somehow help us understand how the geometry of the particles affects the manifestation of the MW effect.

Isolating oblate particles are more successful in making barriers that interrupt the continuity in the permittivity of the background than are prolate particles of the same aspect ratio [7], and most natural granular materials are rather oblate-like [66]. Initially, the effect of changing the associated depolarization from spheres to oblates is to lower the modeled real part of the dielectric response. However, in the case of the SK and PVS mixing models, there are some critical combinations of *a/b*, *ϕ*, *EC_w_* and, for the case of SK, also α, from which a dispersion in the modeled real part below 10^8^ Hz arises, increasing the low-frequency dielectric response. It is also noticeable that these models are very sensitive around this critical set of parameters; small changes in *N^i^* lead to large changes in the modeled real permittivity. From the computations, it has been observed that low-frequency dispersion is favored by the increase in *ϕ*, *EC_w_* and *α* and the approximation of the particles to the oblate shape. Conversely, the more oblate the particles are, the lower the permittivity is in the higher frequency range 10^8^–10^10^ Hz.

**Figure 5:**
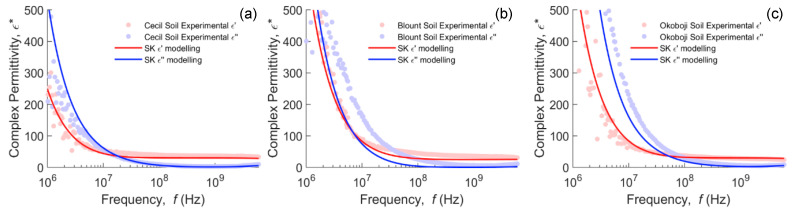
Modeling of the complex dielectric response of Cecil (**a**), Blount (**b**) and Okoboji (**c**) soils using the SK model extended for complex permittivities (Equation (6)) and the extended Debye model (Equation (8)). Optimized parameters were: Cecil (*α* = 0.425, *a/b* = 0.355), Blount (*α* = 0.425, *a/b* = 0.3) and Okoboji (*α* = 0.425, *a/b* = 0.355).

Next, we attempted to fit the SK real permittivity to the data for the three soils by optimizing the heuristic parameter, *α*, and particle depolarization factors, using the least squares method. The imaginary dielectric response was left to float as an independent validation of the ability of the model to correctly predict the imaginary dielectric response with only the real permittivity fitted. The results for the optimum parameters are presented in Figure 5a–c. The fitting was surprisingly consistent with all three soils, returning a value of *α* approximating 0.425 (equivalent to the permittivity resulting from Sen’s model, Equation (3), for spheres) and depolarization factors of 0.30–0.355. If the value for *a/b* is constrained to 0.125, which is more physically realistic for clay tactoids [74], then the heuristic parameter, *α*, is approximately 0.2. The imaginary dielectric response, which was also an optimized object above, corresponded closely with the data measured for the imaginary dielectric response in Cecil and Okoboji samples, showing a shift below 10^9^ Hz in the Blount soil. This was a little surprising as *α* of 0.2 described the effective static permittivity of coarse media with a lower porosity of 0.4 [66], so we were expecting *α* to be similar (or even smaller) for high-porosity soils (0.56 m^3^ m^−3^), but a simple cubic packing of spheres shows this is not necessarily the case (*ϕ* = 0.476 m^3^ m^−3^; *α* = 0.323). However, this finding supports that of Chen and Or [46], who found DEMA suitable for predicting the permittivity of medium-textured soils. It may suggest that the perturbation of the electrical field around an inclusion due to the particle size distribution is sufficient to manifest as an increase in *α*, even at the higher porosity. The shift between the modeled and experimental imaginary permittivity of the Blount sample can be linked to the possibility of an additional dispersion mechanism, i.e., different from conductivity and MW mechanisms.

Other noticeable features include the dispersion in the 10^8^–10^9^ Hz range for all soils, sloping gently from left to right. One thought is that the effect is due to confined water (B in Figure 1, or the microscopic confinement due to caging as outlined in the Introduction), broadening the relaxation of the water phase, a hypothesis consistent with the findings of Calvet [75] (Figure S2) for the first layers of water in homo-ionic montmorillonites. Another dispersion mechanism candidate is the frequency-dependent surface conductivity (S in Figure 1). The relaxation contribution, disregarding the bulk conductivity contribution to the imaginary permittivity, is presented in the Supplementary Material, Figures S3 and S4.

The modeling approach used was found limited at some combinations of the model parameters. The two-phase SK model contains a heuristic parameter, meaning it is not an exact solution of the Laplace equation and therefore can diverge for certain parameter values, hence limiting the feasible solutions. Additionally, as a further exploration of the data, the expected dielectric bounds with a change of bulk density for coarse-textured media are compared with data for talc and the three clayey soils for varying electromagnetic frequencies in Figure S5. The effect of bulk density and electromagnetic frequency is individually presented in Figures S6 and S7, respectively. As a complement to this article, an exploration of the limitations of the models is provided in the Supplementary Material (Figure S8). 

### 4.3. Comparison of Media with Confined Water

**Figure 6:**
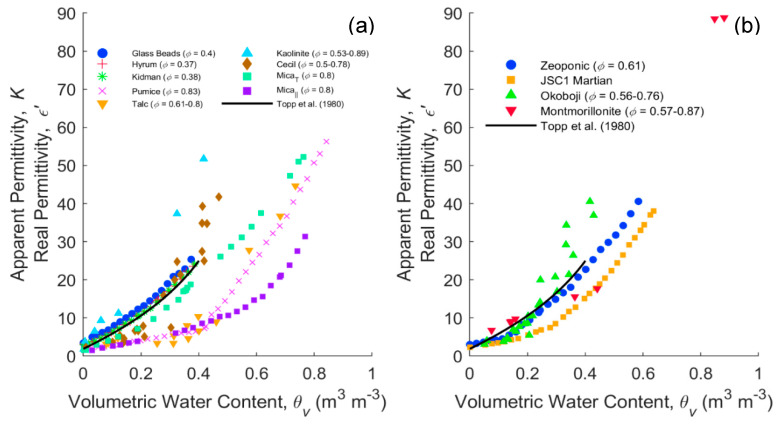
Dielectric (K, ε’)—volumetric water content measurements of different porous media containing negligible (**a**) and substantial (**b**) hygroscopically adsorbed water. Glass Beads, Hyrum and Kidman are from Robinson et al. [77]; Pumice and Zeoponic data from Blonquist et al. [12]; Mica_⊤_ (oblate principal axis perpendicular to the applied electrical field) and Mica_‖_ (oblate principal axis parallel to the applied electrical field) data from Blonquist et al. [76]; JSC1 Martian from Robinson et al. [78]; and Talc, Kaolinite, Cecil, Okoboji and Montmorillonite are all VNA measurements at 10^8^ Hz.

Figure 6 is a compilation of apparent and real permittivity data for different porous media, mostly previously measured using TDR, but also some frequency-domain measurements (10^8^ Hz) from the present study. Figure 6a shows the dielectric response of porous media without microscopically confined water that is electrostatically “bound” (hygroscopically adsorbed) (<0.01 g/g), whereas Figure 6b shows media that have substantial hygroscopic water (>0.04 g/g). Hygroscopic water contents of some media are reported in Table S2, Supplementary Material. In Figure 6a, the glass beads, sandy soils and Topp’s curve provide familiar references. The data of mica from Blonquist et al. [76] show the dielectric response due to the platy (oblate) particle shape of a layered sample that is aligned with or perpendicular to the applied electrical field. Pumice is presented from Blonquist et al. [12], which has a foam-like structure and high porosity. In addition, we have added kaolinite, kaolinitic Cecil soil and talc, but it is important to remember that these materials are repacked, so the geometry changes with each packing. Contrasting responses are observed, with talc having a very low dielectric response and kaolinite, with a similar mineral structure to talc, giving higher dielectric responses than Topp’s curve. We propose that the lower dielectric response of talc could be due to one of a number of mechanisms. The fact that it sits between the aligned and perpendicular mica at water contents beyond ~0.4 might indicate that it has something to do with the alignment of the tactoids with respect to the electrical field. Both kaolinite and talc are very platy materials and follow the same path as mica until a water content of ~0.2 for kaolinite and 0.4 for talc. One might expect the clays to pack perpendicular to the VNA fringing field initially, i.e., the principal axis of the ellipsoid parallel to the applied field, generating more of an open house of cards structure as they become wetter. Second, it is feasible that talc forms microaggregates and macroscopically confines the water as it is a very high porosity material. Thirdly, the clay mineral may microscopically cage the water, confining it, but both kaolinite and talc are relatively low surface area materials, so this is less likely. The fact that kaolinite produces a relatively high dielectric response must be due to the presence of the counter ions, giving rise to a substantial MW relaxation, as shown in Figure 2c. The change in the alignment of the particles is perhaps more consistent with the high-frequency response.

Figure 6b shows the materials with greater amounts of hygroscopic water (>0.04 g/g). Surprisingly, these materials are much closer to Topp’s curve than the materials without hygroscopic water (Figure 6a). Montmorillonite is just under Topp’s curve at a water content of ~0.2, then rises above as others have seen. The simulant JSC1 Martian is a palagonite with a substantial internal surface, but also with substantial hygroscopic water. Zeoponic is an interesting material; unlike clays, it has a granular spherical shape with internal porosity and has the most hygroscopic water. Yet it sits almost on Topp’s curve, contrary to the thesis that microscopically confined water, electrostatically adsorbed, always reduces the dielectric response. This is perhaps one of the most convincing pieces of evidence of the importance of soil structure and the role of confined water.

The evidence presented is not definitive and opens new questions. However, it points to some interesting future potential avenues of research to be carried out. Given we now have a range of microporous media of differing particle sizes, shapes, alignments and surface charges, including nanoporous media, it would be well worth experimenting with these under controlled conditions to explore the role of both the geometry and macro- and micro-confinement of water. In addition, the frequency domain offers a range of opportunities to probe materials and test theories and models. Clearly, there is still a need to develop a general three-phase MHz–GHz dielectric model using an effective medium mixing approach.

## 5. Conclusions

Complex and apparent permittivity data presented for clay minerals and clayey soils demonstrate the importance of MW relaxation processes, increasing permittivity from the high-frequency end to that of the low-frequency end, and yielding a permittivity enhancement that even exceeds the sum of the individual permittivities of the soil phases for water-saturated and nearly water-saturated samples. The extended SK model indicates that the real permittivity response to changes in the depolarization reaches a sensitive point where small changes lead to big changes in permittivity. This point is influenced by the heuristic parameter (*α*), porosity (*ϕ*), *EC_w_* and contrast of conductivity and permittivity between phases. The MG and MWBH models were found inadequate to describe the strong dispersion associated with MW polarization in water-saturated clayey soils. In contrast, models that allow the inclusions to interact strongly, such as PVS (asymmetric effective medium approximation) and SK, were able to approximate the permittivity dispersion below 100 MHz. Fitting the SK model to the data resulted in a value of the heuristic parameter *α*, that accounts for particle interaction, of 0.425 with particle shape depolarization factors surprisingly consistent, with aspect ratios around 3:1, not as extreme as those reported for clay minerals in the literature, but being consistent with an averaged aspect ratio determined by the presence of also other more rounded particles in the soil. Our results suggest that MW relaxation processes are dominant in clayey soils, for example, the kaolinitic soil shows strong dispersion but has negligible microscopically confined water. Data presented suggest that phase composition (porosity and water content) and geometry (particle shape, orientation and size distribution) are the major factors determining the dielectric response and that confined or bound water is secondary.

## Supplementary Materials

The following are available online at https://www.mdpi.com/1424-8220/20/22/6678/s1, Figure S1: Complex permittivity of wet montmorillonites saturated with Na, K, Ca and Mg cations and bulk water, from Cole–Cole parameters in Sposito and Prost [24] (Table III); Figure S2: Dielectric dispersion of water adsorbed on Ca-saturated montmorillonites at different gravimetric water contents and bulk water, from Cole–Cole parameters in Calvet [75]; Figure S3: Bulk conductivity (green) and relaxation (red) contributions to the measured imaginary permittivity (black) in the water-saturated Cecil (a), Blount (b) and Okoboji (c) soils; Figure S4: Normalized relaxation contribution to the imaginary permittivity in the water-saturated Cecil, Blount and Okoboji soils; Figure S5: Real permittivity bounds for glass beads (light gray) and quartz sand (medium gray) (the dark gray-colored region is their overlap), using the layer model (Equation (S4)) [77] with SK (Equation (6)) for dry and water-saturated states, compared to talc (a), Cecil kaolinitic (b), Blount illitic (c) and Okoboji montmorillonitic (d) soils experimental data. The porosity of the different samples is marked with arrows. Also shown for comparison are the empirical calibrations presented by Topp et al. [1] for glass beads and Rubicon sandy loam soil;; Figure S6: Forward modeling of the expected porosity-dependent response of unsaturated glass spheres (a) and quartz sand (b) as a function of water content using the layer model (Equation (S4)) with the Sihvola–Kong (Equation (6)) and Debye models (Equation (7), *f* = 250 MHz) with Kaatze [70] water permittivity at 25 °C. Glass beads (porosity, ϕ ~ 0.395; aspect ratio, a/b ~ 1), quartz sand (ϕ ~ 0.382; a/b ~ 0.466). α = 0.2 in both cases; Figure S7: Forward modeling of the expected frequency-dependent response of unsaturated glass spheres (a) and quartz sand (b) as a function of water content using the layer model (Equation (S4)) with the Sihvola–Kong (Equation (6)) and Debye models (Equation (7)) with Kaatze [70] water permittivity at 25 °C. Glass beads (porosity, ϕ ~ 0.395; aspect ratio, a/b ~ 1), quartz sand (ϕ ~ 0.382; a/b ~ 0.466). α = 0.2 in both cases; Figure S8: (a) Inconsistent solutions (colored volume) of SK for a two-phase water (*ε* ~ 80)–solid mixture (ε ~ 5) as a function of the aspect ratio, heuristic parameter and inclusions volumetric fraction; (b) 2D representation of (a) for any value of f, including the heuristic parameter value for the equivalence with the well-known MG, PVS and CP models Table S1: Mineralogy, particle size and particle density of mineral samples (textbook values of particle density from Weast et al. [79]); Table S2: Values of hygroscopic water measured in mineral and mineral soil samples.
