# Supplementary material for: Dielectric Spectroscopy and Application of Mixing Models Describing Dielectric Dispersion in Clay Minerals and Clayey Soils
Source: Sensors (Basel). 2020 Nov 22;20(22):6678. doi: 10.3390/s20226678 (PMC7700415; doi:10.3390/s20226678)
Supplement: Supplementary file 1 [file sensors-20-06678-s001.pdf]

Supplementary Material

Dielectric Spectroscopy and Application of Mixing Models Describing Dielectric Dispersion in Clay Minerals and Clayey Soils

Juan D. González-Teruel [1,*], Scott B. Jones [2], Fulgencio Soto-Valles [1], Roque Torres-Sánchez [1], Inmaculada Lebron [3], Shmulik P. Friedman [4] and David A. Robinson [2,3]

1 Department of Automatics, Electrical Engineering and Electronic Technology, Technical University of Cartagena, 30202 Murcia, Spain; pencho.soto@upct.es (F.S.-V.); roque.torres@upct.es (R.T.-S.)
2 Department Plants, Soils and Climate, Utah State University, Logan, UT 84322, USA; scott.jones@usu.edu (S.B.J.); davi2@ceh.ac.uk (D.A.R.)
3 UK Centre for Ecology and Hydrology, ECW, Bangor LL572UW, UK; inmbin@ceh.ac.uk
4 Institute of Soil, Water and Environmental Sciences, Agricultural Research Organization, The Volcani Center, Rishon LeZion 7505101, Israel; vwsfried@volcani.agri.gov.il
* Correspondence: juando.gonzalez@upct.es; Tel.: +34-968-32-53-92

Received: 13 October 2020; Accepted: 6 November 2020; Published: date

Clay Minerals Properties

Table S1. Mineralogy, particle size and particle density of mineral samples (textbook values of particle density from Weast et al. [1]). The clays used were standards purchased from the Clay Mineral Society, associated physical and chemical data can be found at: http://www.clays.org/sourceclays_data.html.

**Mineral**	**Composition**	**Structure**	**Denomination and Particle Size**	**Particle density g cm^{-3} (measured)**	**Particle density g cm^{-3} (Literature value range)**
Quartz sand			500 µm	2.60	2.65
Glass			500 µm	2.71	
Talc, Sigma Aldrich standard	hydrous magnesium silicate	1:1 trioctahedral	2-20 µm	2.70	2.58 - 2.83
Kaolinite Clay Mineral Society standard (KGa-1), Washington County, GA USA.	$Si_2Al_2O_5(OH)_4$	1:1 dioctahedral	<2 µm	2.58	2.61 - 2.68
Illite Clay Mineral Society standard (Imt-2) Silver Hill, Cambrian Shale, USA.	aluminum silicate	2:1 dioctahedral	<2 µm	2.72	2.60 - 2.90
Montmorillonite, Clay Mineral Society standard (SWy-2) Na- Crook County, Wyoming, USA.	Na-aluminum silicate	2:1 dioctahedral	<2 µm	2.67	2 - 3

Hygroscopic Water Measurements

The hygroscopic water content of the minerals and mineral soils was obtained with the method described in [2] at ~20% of relative humidity equivalent to one layer of adsorbed water.

Table S2. Values of hygroscopic water measured in mineral and mineral soil samples.

Mineral /Soil	Hygroscopic water 19% humidity $g_{WATER}\ g_{SOIL}^{-1}$
Quartz sand	0.0002
Glass	0.0001
Talc	0.001
Kaolin	0.0023
Illite	0.0184
Montmorillonite	0.0490
Okoboji	0.05
Pumice	0.0061
Zeoponic	0.047
JSC1 Martian	0.04

Dielectric Behavior of Water Adsorbed on Homoionic Montmorillonites (Literature Data)

Figure S1 presents the dispersion of water adsorbed on Na-, K-, Ca- and Mg-Montmorillonites compared to that of free or bulk water at 20 °C from the Cole-Cole parameters reported in Sposito and Prost [3], Table III. This illustrates the dielectric behavior of bound water in montmorillonites according to the predominant cationic charges presented on the surface, although the MW dielectric relaxation of the univalent clays because of the <1 aspect ratio of the univalent particles (smaller number of platelets within a single tactoid) is likely intensifying the relaxation. The data correspond to gravimetric water contents of 0.06, 0.085, 0.07 and 0.07 g H_2O/g clay for Na-, K-, Ca- and Mg-Montmorillonites, respectively. The relaxation time of water adsorbed on clays saturated with monovalent cations (Na and K) is similar to that of bulk water, but considerably reduced in that saturated with divalent cations (Ca and Mg).

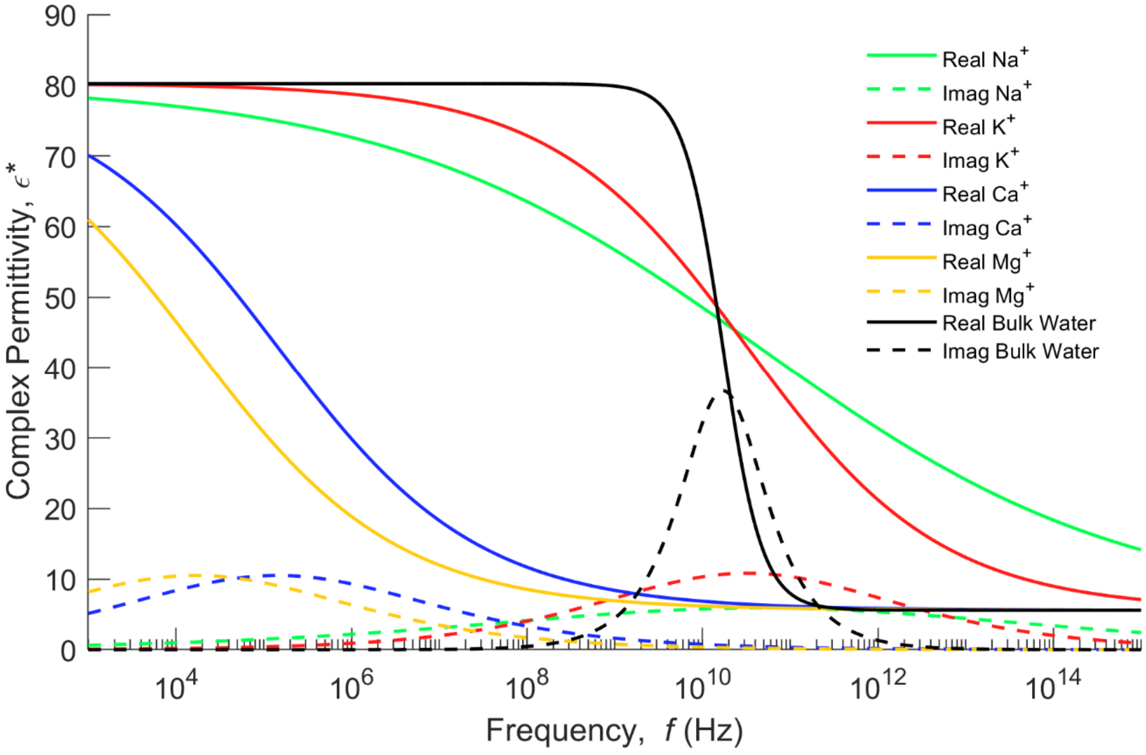

Figure S1. Complex permittivity of wet montmorillonites saturated with Na, K, Ca and Mg cations and bulk water, from Cole-Cole parameters in Sposito and Prost [3], Table III.

Figure S2 shows the dispersion of water adsorbed on Ca-Montmorillonite at different gravimetric water contents compared to that of free or bulk water at 20 °C from the Cole-Cole parameters reported in Calvet [4]. The relaxation time is considerably increased for low water contents (<10 kg H2O/kg clay) and close to that of bulk water beyond. However, the broadened relaxation seems to remain, despite increasing the water content. The non-monotonous behavior of the trend with the water content could indicate that the dispersion results also from the MW relaxation and this is possible because of the complex and interacting MW processes occurring on both the soil-water and water-air interfaces.

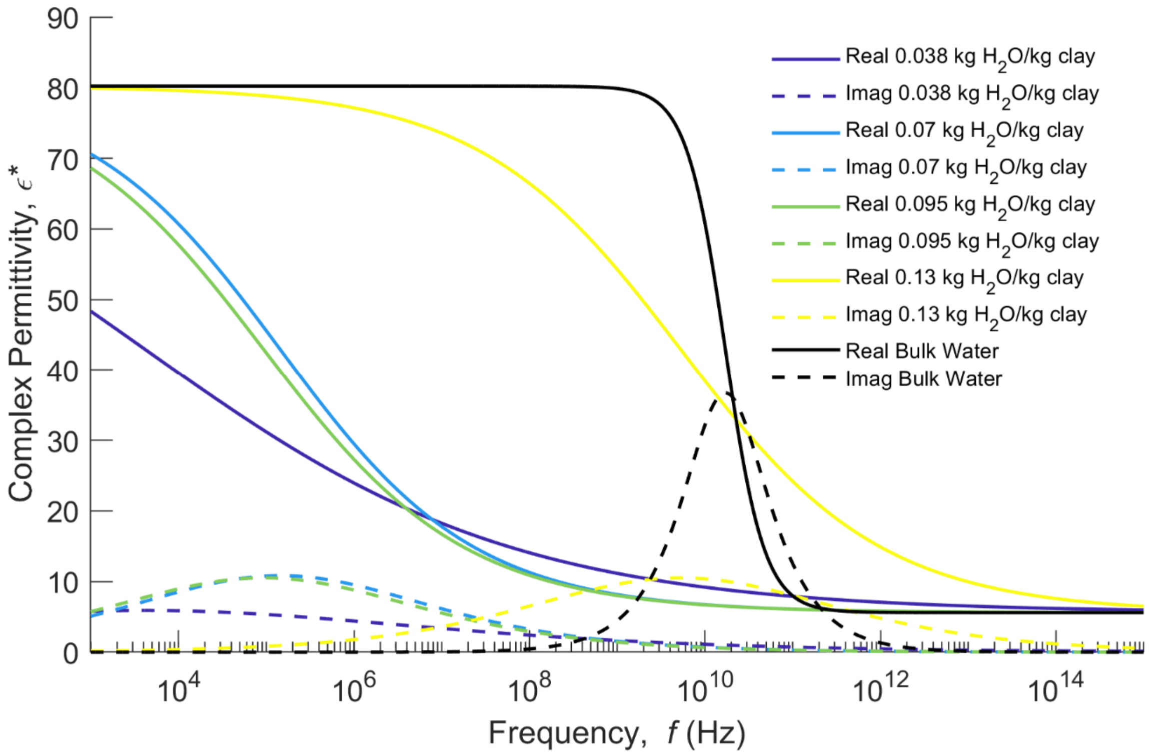

Figure S2. Dielectric dispersion of water adsorbed on Ca saturated montmorillonites at different gravimetric water contents and bulk water, from Cole-Cole parameters in Calvet [4].

Water-Saturated Clayey Mineral Soils, Bulk DC Conductivity

The complex permittivity of a medium can be expressed as:

$$\varepsilon^* = \varepsilon'(\omega) - j\varepsilon''(\omega) = \varepsilon'(\omega) - j\left(\varepsilon''_{rel}(\omega) + \frac{\sigma_{aDC}}{\omega\varepsilon_0}\right) \quad \text{(S1)}$$

where ε′ is the real part, associated with the energy storage and ε′′ is the imaginary part, describing energy losses. The latter is the sum of a relaxation term, ε''_{rel}, and a conductivity term, $\frac{\sigma_{aDC}}{\omega\varepsilon_0}$.

The bulk Direct Current (DC) conductivity, σ_{aDC}, of the water saturated clayey mineral soils samples studied was obtained by fitting the $\frac{\sigma_{aDC}}{\omega\varepsilon_0}$ expression to the measured imaginary permittivity. In a $\log_{10}\frac{\omega}{2\pi} - \log_{10}\varepsilon''$ plot, $\frac{\sigma_{aDC}}{\omega\varepsilon_0}$ is a straight line, which is usually asymptotic to the frequency-dependent imaginary permittivity logarithmic response, σ_{aDC} being a constant. The fitting was performed in the 1 MHz – 100 MHz range, since below 1 MHz the experimental data were noisy and above 100 MHz the imaginary permittivity starts to diverge from linear behavior. In Figure S3 the imaginary permittivity is plotted as a function of frequency in a logarithmic scale. The conductivity contribution, obtained by least squares fitting, and the relaxation contribution are also plotted independently, along with the real part of the permittivity.

In Figure S4, the relaxation contribution is normalized dividing by the total imaginary permittivity, so that its contribution in the different soils can be compared. The Cecil and Blount soils show a relative higher contribution of the relaxation term in the 10^7 – 10^9 bandwidth. Hence, the dispersion in this bandwidth is not as strongly dominated by the *EC* as it is in the Okoboji soil, opening the door to an additional relaxation mechanism apart from the conductivity and MW mechanisms.

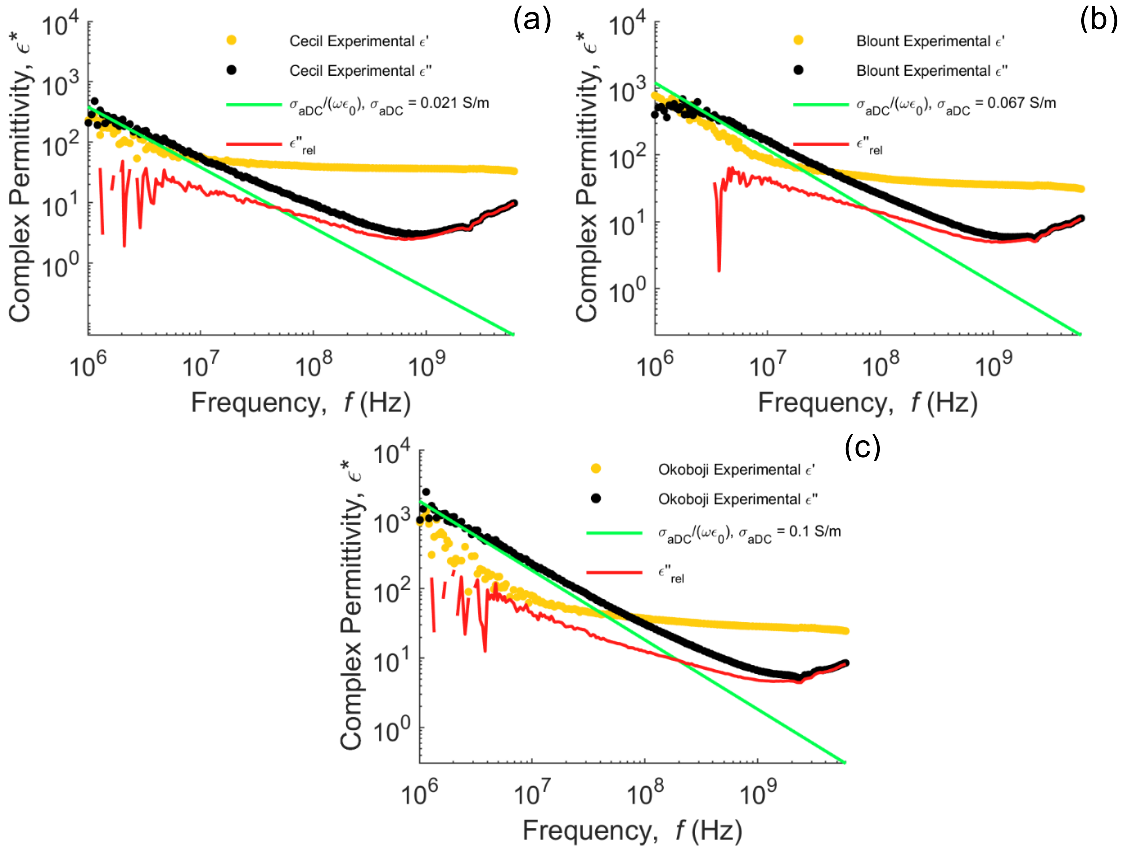

Figure S3. Bulk conductivity (green) and relaxation (red) contributions to the measured imaginary permittivity (black) in the water saturated Cecil (**a**), Blount (**b**) and Okoboji (**c**) soils.

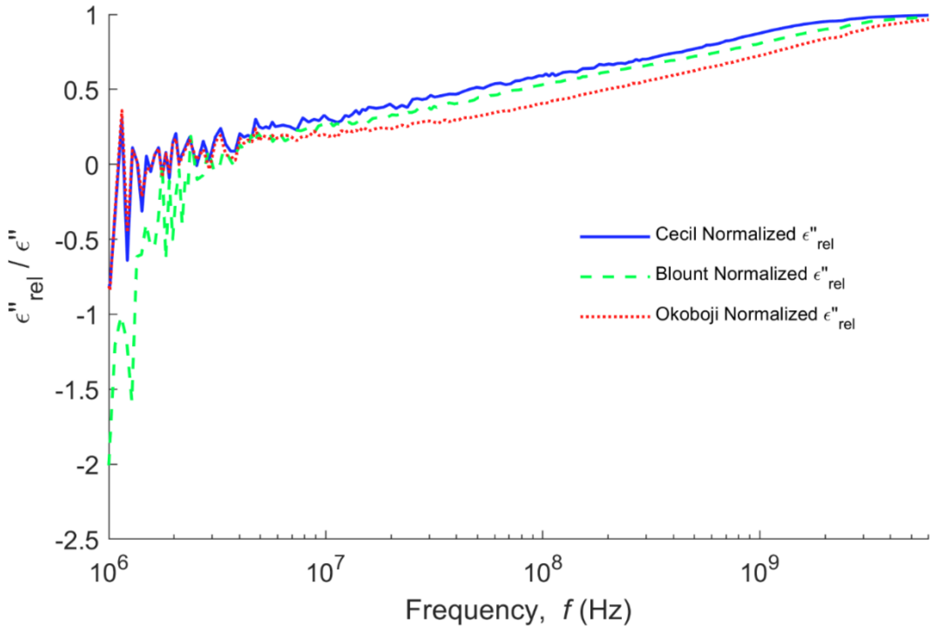

Figure S4. Normalized relaxation contribution to the imaginary permittivity in the water saturated Cecil, Blount and Okoboji soils.

Unsaturated Coarse-Textured Soils Layered Model

Robinson et al. [5] presented a model that treats the porous medium as two dielectric layers composed of 2-phases: air-dry and water-saturated each with an apparent permittivity. Using the

TDR-measured apparent permittivity, *K*, of a layered material, the apparent permittivity for the layers can be derived. The travel time of the TDR signal through layers *L1, L2* with combined length of *L* = *L1*+*L2* is given in Equation S2.

$$t(L_x) = \frac{L_x}{v_p} \propto L_x \sqrt{K_x} \tag{S2}$$

where v_p is the TDR electromagnetic signal propagation velocity in a normal direction to the layers and the subscript x denotes an arbitrary dielectric. Thus, based on square root averaging of the permittivity "refractive index mixture theory", the measured permittivity is:

$$K = \left(\frac{L_1\sqrt{K_1} + L_2\sqrt{K_2}}{L}\right)^2 \tag{S3}$$

This expression has been used previously to model layered soils [6,7]. Work by [8–10] showed that this holds for TDR measurements with relatively thick layers such as for wetting fronts in soils and sediments but not for multiple thin layers. Robinson et al. [5] therefore proposed the following equation to describe a bi-layered wetting or draining soil profile:

$$\sqrt{K} = \sqrt{\varepsilon_{sat}}\left(\frac{\theta}{\varphi}\right) + \sqrt{\varepsilon_{dry}}\left(1 - \frac{\theta}{\varphi}\right) \tag{S4}$$

where, θ is the mean volumetric water content and ϕ is the porosity for all layers. The two, two-phase mixture permittivities of saturated (ε_{sat}) and dry (ε_{dry}) materials can be measured or derived from any two-phase dielectric-mixing model. This results in a calibration model that is porosity dependent and can be fitted to most soils that do not have dielectric dispersion, simply using the permittivity at the saturated moisture content and assuming the permittivity of the dry soil to be ~2.8.

Bulk Density and Frequency Bounds for Unsaturated Soils

As a further exploration of the data, the expected dielectric bounds with a change of bulk density for glass beads and quartz sand are compared with data for talc and the three clay soils for varying electromagnetic frequencies. Multiple ε' - θ_V curves were obtained with porosity varied from 0.3 to 0.75, with 0.0225 steps; and electromagnetic frequency from 10^7 Hz to 6 x 10^9 Hz, with arbitrary steps. The permittivity response envelopes were constructed based on the layer model for unsaturated coarse-textured soils proposed in Robinson et al. [5] (Equation S4), using the SK model (Equation 6) for dry and saturated states. EC_w was set to 0 and α to 0.2. The permittivity of glass beads and quartz sand solid particles was set to 7.6 and 4.7, respectively, as reported in Robinson et al. [5]. The perimeter points of the set of computations constitute the bounds of the permittivity envelopes. In Figure S5 the envelopes correspond to light gray for glass beads and medium gray for quartz sand (the dark gray colored region is their overlap) and are compared with talc (a) and clayey soils experimental data (b-d) at different electromagnetic frequencies. Topp et al. [11] equations for glass beads and Rubicon sandy loam soil are also plotted for comparison. In Figures S6 and S7, porosity and frequency effects are presented independently.

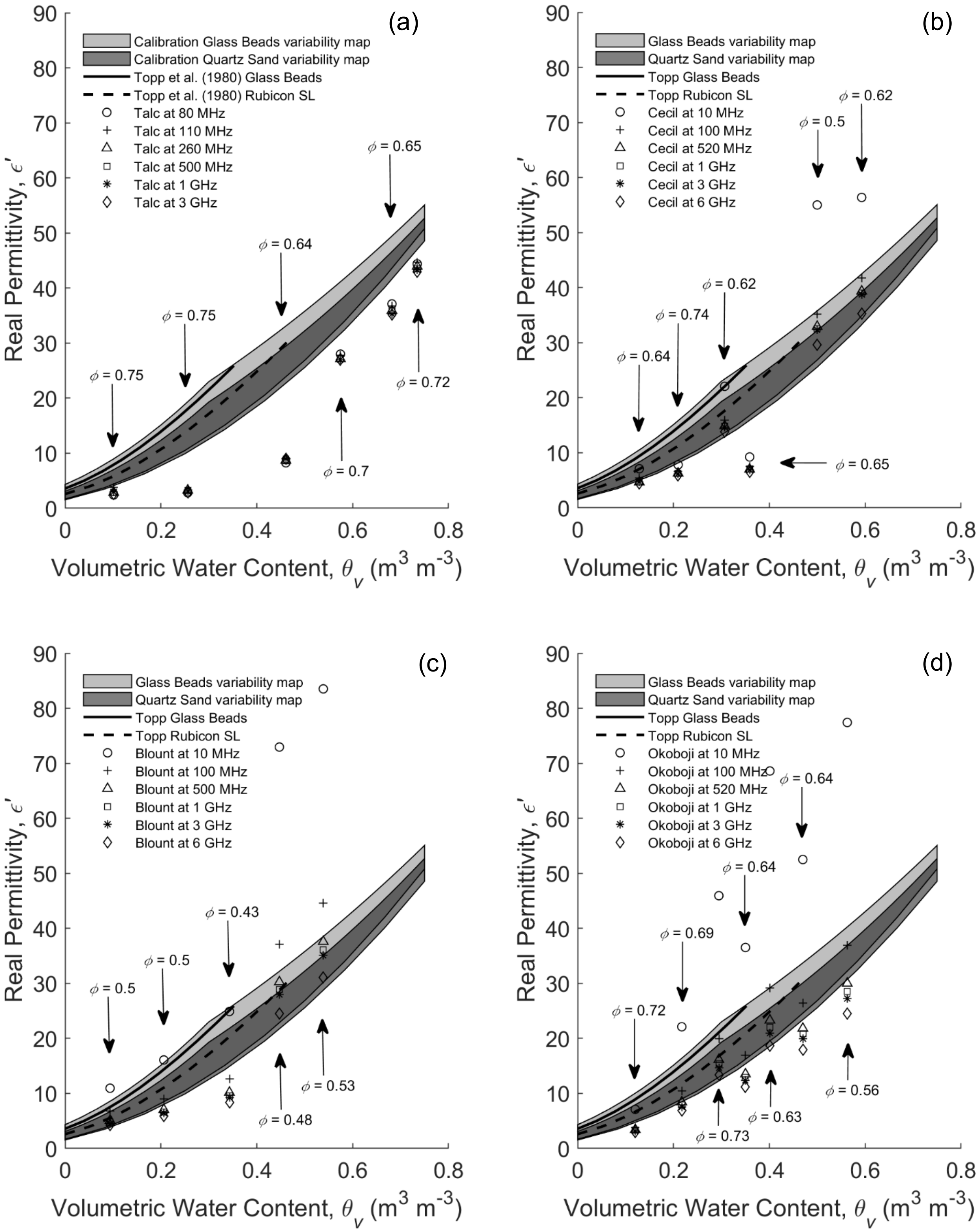

Figure S5. Real permittivity bounds for glass beads (light gray) and quartz sand (medium gray) (the dark gray colored region is their overlap), using the layer model (Equation S4) [5] with SK (Equation 6) for dry and water-saturated states, compared to talc (a), Cecil kaolinitic (b), Blount illitic (c) and Okoboji montmorillonitic (d) soils experimental data. The porosity of the different samples is marked with arrows. Also shown for comparison are the empirical calibrations presented by Topp et al. [11] for glass beads and Rubicon sandy loam soil.

If the clay mineral permittivity response was simply due to changes in bulk density (porosity), we would expect the experimental data to fit within these envelopes (as in Friedman [12]). Clearly all soil samples are to some extent outside of the envelopes. The talc (a) falls consistently below the envelopes for all data. The theory that this low permittivity response is due to the presence of bound water is not plausible, as talc has none, and opens the door to other mechanisms, such as phase configuration confining water as talc can aggregate [13,14]. The three clayey soils fall below the envelope initially, but as the water content increases, and consequently also the bulk electrical conductivity, the dielectric response increases, so that in the case of all three soils it rises above the envelope for wet soils at low frequencies. The lower permittivity values at high frequencies are consistent with TDR literature measurements [15–17]. The high values at low frequencies are consistent with the findings of [18], who also found high values of permittivity for clayey soils using a 20MHz sensor. These findings are all consistent with our modelling and that of Chen and Or [19] showing that Maxwell Wagner phenomena exert a dominant influence over the permittivity response of soils in the MHz-GHz frequency bands. We suggest that future permittivity-water content calibration models must explore and account for these phenomena to make progress in understanding. Moreover, when reporting results from new sensors, the results here show evidence for the necessity of specifying the electromagnetic effective frequency for which the measurements were made to understand potential dispersion effects, as the dielectric response can vary widely in clayey soils and when *EC* is significant.

Estimated Porosity-Dependent Dielectric Response of Glass Spheres and Quartz Sand

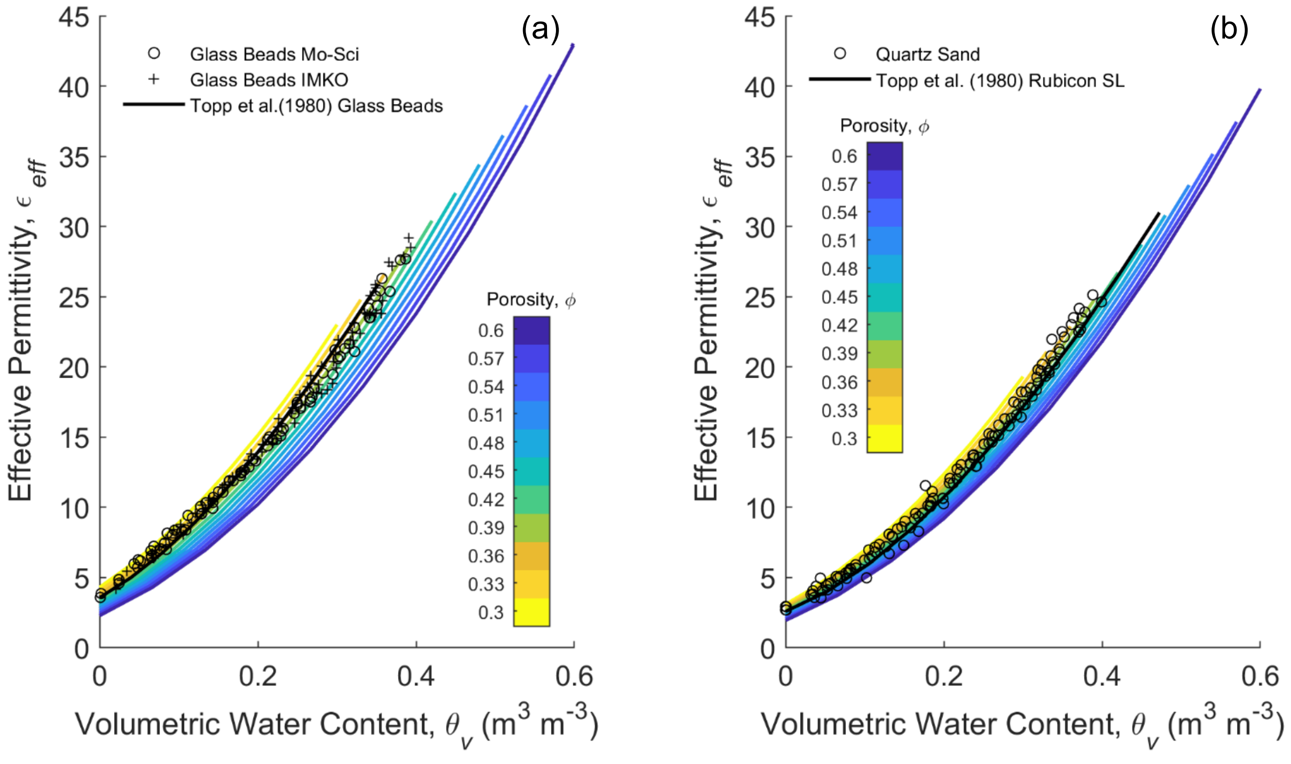

Figure S6. Forward modelling of the expected porosity-dependent response of unsaturated glass spheres (a) and quartz sand (b) as a function of water content using the layer model (Equation S4) with Sihvola-Kong (Equation 6) and Debye model (Equation 7, f = 250 MHz) with Kaatze [21]water permittivity at 25°C. Glass beads (porosity, ϕ ~ 0.395; aspect ratio, a/b ~ 1), quartz sand (ϕ ~ 0.382; a/b ~ 0.466). α = 0.2 in both cases.

Estimated Frequency-Dependent Dielectric Response of Glass Spheres and Quartz Sand

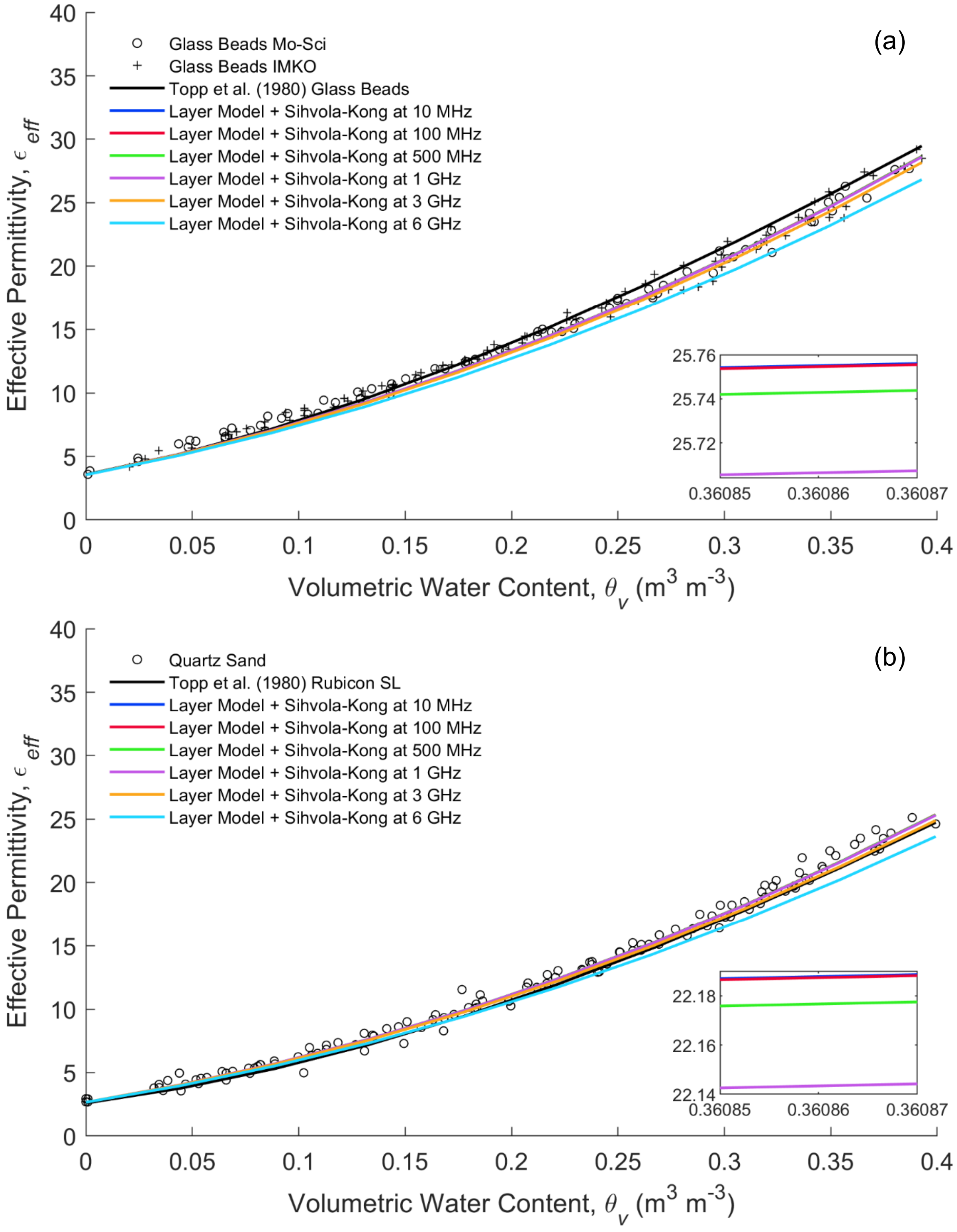

Figure S7. Forward modelling of the expected frequency-dependent response of unsaturated glass spheres (**a**) and quartz sand (**b**) as a function of water content using the layer model (Equation S4) with Sihvola-Kong (Equation 6) and Debye model (Equation 7) with Kaatze [21] water permittivity at 25°C. Glass beads (porosity, ϕ ~ 0.395; aspect ratio, a/b ~ 1), quartz sand (ϕ ~ 0.382; a/b ~ 0.466). α = 0.2 in both cases.

Limitations of the Modelling Approaches

It is important to point out some of the limitations of the modelling approaches used. The 2-phase SK model contains a heuristic parameter, meaning it is not an exact solution of the Laplace equation and therefore can diverge for certain parameter values, hence limiting the feasible solutions. Since we found that the model provided physically 'inconsistent' solutions, i.e. $\varepsilon' < 0$ or $\varepsilon'' > 0$, a 'consistency of boundaries' set of computations of the model was performed, although for convenience, only for the static case, for which only real solutions are expected. A water background ($\varepsilon \sim 80$) – solid inclusions ($\varepsilon \sim 5$) mixture configuration was considered, where the heuristic parameter (α), aspect ratio (a/b) and inclusion volumetric fractions (f) ranged from 0 – 1, 10^{-3} – 10^{3} and 0 – 1, respectively. 'Inconsistent' solutions were considered as those either lower than the permittivity of the solid inclusions or with a non-zero imaginary result. Additionally, we found solutions out of the 'inconsistency' boundaries defined, but with non-physical sense, showing a steep fall in effective permittivity (> 25) for increases of f values lower than 0.1. These solutions were also considered as 'inconsistent' and were found for high interactions between particles (α near 1) and f values around 1. In Figure S8a the colored area represents the α, a/b and f combinations for which the model does not yield 'consistent' solutions. Figure S8b represents a two-dimensional view of Figure S8a, including only the α and a/b axis. The model is clearly limited as the value of α increases, especially for oblate spheroids, as the more oblate the inclusions are, the fewer are the 'consistent' solutions. For prolate spheroids there is also an affected area, although only for values of α close to 1, where the model provides an unrealistic approximation. Jones and Friedman [14] showed realistic convergence of the model for high enough α values for low contrast in permittivity between the two phases (solid-air configuration, 5:1, characteristic of dry soil), but here divergence issues yield for high contrast (solid-water configuration, 5:80, characteristic of water-saturated soil). The SK model was also found problematic when dealing with complex values, since it results in a third degree equation, giving rise to three possible solutions. Sometimes two of the solutions can be discarded because they are physically 'inconsistent', yet when this is not the case, it is not trivial to make a choice.

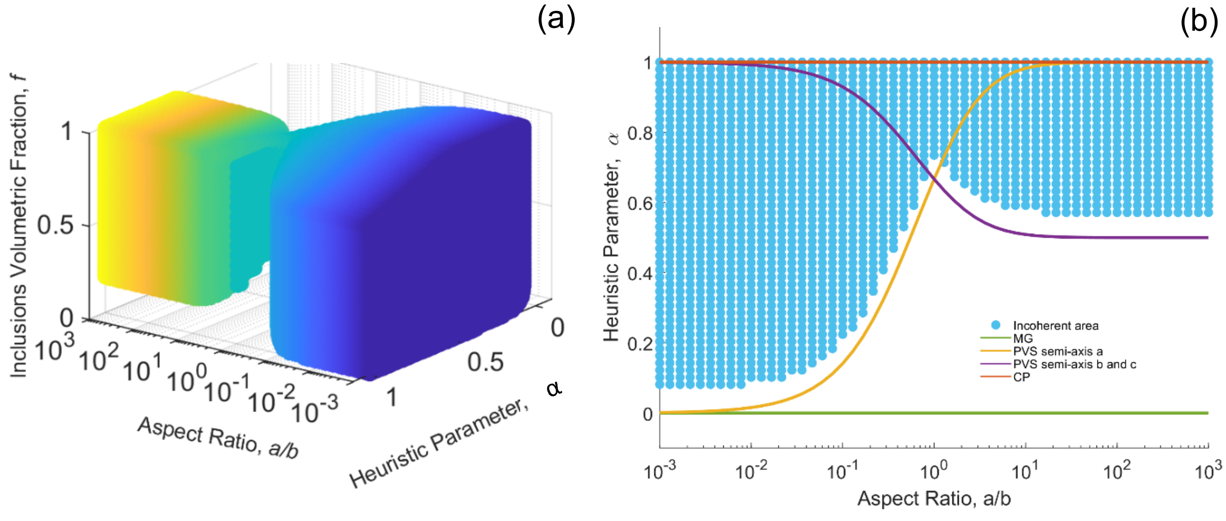

Figure S8. (a) Inconsistent solutions (colored volume) of SK for a 2-phase water ($\varepsilon \sim 80$) – solid mixture ($\varepsilon \sim 5$) as a function of the aspect ratio, heuristic parameter and inclusions volumetric fraction; (b) 2D representation of (a) for any value of f, including the heuristic parameter value for the equivalence with the well-known MG, PVS and CP models.

The bi-layer modelling approach used for Figure S5 addresses only a layered system where one layer is practically water-saturated and the other close to air-dry. First, the model is designed for a steep wetting/drying front (likely to be encountered in coarse-textured media) along the length of the sensor electrodes and does not describe a continuously distributed water profile. Second, the model does not currently describe soils with bound water but the data here suggest the role of bound water is secondary in these media compared to the impact of geometry and dispersion processes. In order

to extend the modelling to describe a partially-saturated soil within a distributed water profile, two additional extension steps are required: (i) replace the refractive index averaging with an effective medium theory that depends on the steepness of the wetting front (correlation length of the water content) and the dominant wavelength [10]. This might be achieved with the derivation of a proper pore-scale εeff (θ_V) model following, for example, the approach presented in Friedman [20] which would allow different configurations of the air/water phases. To tackle the second, we still need to understand the relative contribution of geometry and confined water to the effective permittivity.

References

1. Weast, R.C.; Astel, M.J.; Beyer, W.H. Handbook of Chemistry and Physics. 67th ed.CRC Press Inc: Boca Ratón, Florida, 1986.
2. Quirk, J.P.; Murray, R.S. Appraisal of the Ethylene Glycol Monoethyl Ether Method for Measuring Hydratable Surface Area of Clays and Soils. Soil Sci. Soc. Am. J. 1999, 63, 839–849.
3. Sposito, G.; Prost, R. Structure of Water Adsorbed on Smectites. Chem. Rev. 1982, 82, 553–573.
4. Calvet, R. Absorption dipolaire et conductivité de l'eau adsorbée sur la montmorillonite calcique. In Proceedings of the International Clay Conference; Madrid, 1972; pp. 2:519-528.
5. Robinson, D.A.; Jones, S.B.; Blonquist, J.M.; Friedman, S.P. A Physically Derived Water Content/Permittivity Calibration Model for Coarse-Textured, Layered Soils. Soil Sci. Soc. Am. J. 2005, 69, 1372–1378.
6. Birchak, J.R.; Gardner, C.G.; Hipp, J.E.; Victor, J.M. High Dielectric Constant Microwave Probes for Sensing Soil Moisture. Proc. IEEE 1974, 62, 93–98.
7. Topp, G.C.; Davis, J.L.; Annan, A.P. Electromagnetic determination of soil water content using TDR: I. Applications to wetting fronts and steep gradients. Soil Sci. Soc. Am. J. 1982, 46, 672–678.
8. Chan, C.Y.; Knight, R.J. Determining water content and saturation from dielectric measurements in layered materials. Water Resour. Res. 1999, 35, 85–93.
9. Chan, C.Y.; Knight, R.J. Laboratory measurements of electromagnetic wave velocity in layered sands. Water Resour. Res. 2001, 37, 1099–1105.
10. Schaap, M.G.; Robinson, D.A.; Friedman, S.P.; Lazar, A. Measurement and modeling of the TDR signal propagation through layered dielectric media. Soil Sci. Soc. Am. J. 2003, 67, 1113–1121.
11. Topp, G.C.; Davis, J.L.; Annan, A.P. Electromagnetic determination of soil water content: Measurements in coaxial transmission lines. Water Resour. Res. 1980, 16, 574–582.
12. Friedman, S.P. Statistical mixing model for the apparent dielectric constant of unsaturated porous media. Soil Sci. Soc. Am. J. 1997, 61, 742–745.
13. Blonquist, J.M.; Jones, S.B.; Lebron, I.; Robinson, D.A. Microstructural and phase configurational effects determining water content: Dielectric relationships of aggregated porous media. Water Resour. Res. 2006, 42.
14. Jones, S.B.; Friedman, S.P. Particle shape effects on the effective permittivity of anisotropic or isotropic media consisting of aligned or randomly oriented ellipsoidal particles. Water Resour. Res. 2000, 36, 2821–2833.
15. Bridge, B.J.; Sabburg, J.; Habash, K.O.; Ball, J.A.R.; Hancock, N.H. The dielectric behaviour of clay soils and its application to time domain reflectometry. Aust. J. Soil Res. 1996, 34, 825–835.
16. Ponizovsky, A.A.; Chudinova, S.M.; Pachepsky, Y.A. Performance of TDR calibration models as affected by soil texture. J. Hydrol. 1999, 218, 35–43.
17. Dirksen, C.; Dasberg, S. Improved Calibration of Time Domain Reflectometry Soil Water Content Measurements. Soil Sci. Soc. Am. J. 1993, 57, 660–667.
18. Perdok, U.D.; Kroesbergen, B.; Hilhorst, M.A. Influence of gravimetric water content and bulk density on the dielectric properties of soil. Eur. J. Soil Sci. 1996, 47, 367–371.
19. Chen, Y.; Or, D. Effects of Maxwell-Wagner polarization on soil complex dielectric permittivity under variable temperature and electrical conductivity. Water Resour. Res. 2006, 42.
20. Friedman, S.P. A saturation degree-dependent composite spheres model for describing the effective dielectric constant of unsaturated porous media. Water Resour. Res. 1998, 34, 2949–2961.
21. Kaatze, U. Complex permittivity of water as a function of frequency and temperature. J. Chem. Eng. Data 1989, 34, 371–374.
